# African Trypanosomiasis-Associated Anemia: The Contribution of the Interplay between Parasites and the Mononuclear Phagocyte System

**DOI:** 10.3389/fimmu.2018.00218

**Published:** 2018-02-15

**Authors:** Benoit Stijlemans, Patrick De Baetselier, Stefan Magez, Jo A. Van Ginderachter, Carl De Trez

**Affiliations:** ^1^Laboratory of Cellular and Molecular Immunology, Vrije Universiteit Brussel (VUB), Brussels, Belgium; ^2^Myeloid Cell Immunology Laboratory, VIB Center for Inflammation Research, Brussels, Belgium; ^3^Laboratory for Biomedical Research, Ghent University Global Campus, Incheon, South Korea

**Keywords:** anemia, MPS, MIF, erythrophagocytosis, inflammation, hemodilution, IL-10, IFN-γ

## Abstract

African trypanosomosis (AT) is a chronically debilitating parasitic disease of medical and economic importance for the development of sub-Saharan Africa. The trypanosomes that cause this disease are extracellular protozoan parasites that have developed efficient immune escape mechanisms to manipulate the entire host immune response to allow parasite survival and transmission. During the early stage of infection, a profound pro-inflammatory type 1 activation of the mononuclear phagocyte system (MPS), involving classically activated macrophages (i.e., M1), is required for initial parasite control. Yet, the persistence of this M1-type MPS activation in trypanosusceptible animals causes immunopathology with anemia as the most prominent pathological feature. By contrast, in trypanotolerant animals, there is an induction of IL-10 that promotes the induction of alternatively activated macrophages (M2) and collectively dampens tissue damage. A comparative gene expression analysis between M1 and M2 cells identified galectin-3 (Gal-3) and macrophage migration inhibitory factor (MIF) as novel M1-promoting factors, possibly acting synergistically and in concert with TNF-α during anemia development. While Gal-3 enhances erythrophagocytosis, MIF promotes both myeloid cell recruitment and iron retention within the MPS, thereby depriving iron for erythropoiesis. Hence, the enhanced erythrophagocytosis and suppressed erythropoiesis lead to anemia. Moreover, a thorough investigation using MIF-deficient mice revealed that the underlying mechanisms in AT-associated anemia development in trypanosusceptible and tolerant animals are quite distinct. In trypanosusceptible animals, anemia resembles anemia of inflammation, while in trypanotolerant animals’ hemodilution, mainly caused by hepatosplenomegaly, is an additional factor contributing to anemia. In this review, we give an overview of how trypanosome- and host-derived factors can contribute to trypanosomosis-associated anemia development with a focus on the MPS system. Finally, we will discuss potential intervention strategies to alleviate AT-associated anemia that might also have therapeutic potential.

## Introduction

African trypanosomes are extracellular protozoan parasites causing debilitating diseases of medical, veterinary, and socioeconomical importance that adversely affect the economic development of sub-Saharan Africa (1–3). The distribution of the disease coincides with the habitat of the tsetse fly vector (*Glossina* spp.), and is called the tsetse fly “belt” or is sometimes referred to as “green desert” due to the fact that ~10 million km^2^ of potential fertile land is rendered unsuitable for cultivation (3). Within this area, the majority of the 39 tsetse-infested countries are underdeveloped, poor, heavily indebted, food-deficit countries due to the lack of productive animals as far as meat/milk production and draft power are concerned, resulting in an annual economic loss of about 5 billion US$ (4, 5). In addition, about 60 million people living in this belt are at potential risk of infection with an estimated mortality rate of about 10,000 per year (6). Due to the low incidence of African trypanosomiasis, it is also considered a neglected disease. The disease caused by these extracellular hemoflagellates in humans is known as “sleeping sickness” or human African trypanosomiasis (HAT), while in domestic animals it is called “nagana” or animal African trypanosomiasis (AAT) (7). As far as HAT is concerned, two distinct subspecies of *Trypanosoma brucei* are responsible for the disease: (i) *Trypanosoma brucei gambiense*, typically found in western and central Africa (representing 98% of all cases, with humans as main reservoir), causes a chronic form of HAT (a few months to over several years) and (ii) *Trypanosoma brucei rhodesiense*, found in eastern and southern Africa [representing about 2% of all HAT cases due to the fact it is a zoonosis form with animals as main reservoir and humans being occasionally infected (8, 9)], generally causes an acute form of HAT leading to death within a few months if left untreated (6, 10, 11). HAT is characterized by two successive stages: an early hemolymphatic stage, whereby the parasites are observed in the peripheral blood and the lymphatic system, and a later meningoencephalitic stage, where parasites cross the blood–brain barrier and proliferate in the cerebral spinal fluid resulting in neurological complications/cerebral pathology and death if left untreated (12, 13). As far as AAT is concerned, the strictly intravascular parasites *Trypanosoma congolense*, as well as *Trypanosoma vivax*, can be considered the most important causative agents (14). Yet, also *Trypanosoma brucei brucei* and *Trypanosoma evansi*, residing both in intravascular as well as extravascular spaces within their host, have been documented to contribute to livestock infections (14–16). In contrast to game animals, where these parasites cause only mild infections, the disease in domestic animals is severe and often fatal (5, 17, 18).

Various methods have been implemented to control African trypanosomiasis (19); including (i) vector control (20), (ii) reducing the proximity of livestock to reservoir hosts, (iii) development op trypanotolerant livestock (disease-resistant breeds) (5, 21), and (iv) using trypanocidal drugs (22). Yet, their success is limited due to the fact that these techniques are often used locally and not necessarily in a coordinated fashion (23), game animals function as parasite reservoir without exhibiting pathological signs (24), and the rapid emergence of drug-resistant trypanosomes, thereby undermining their efficacy and leading to the widespread outbreaks of trypanosomiasis (19, 25, 26).

The main factor hampering control over African trypanosomiasis is the fact that these parasites have evolved very efficient immune escape mechanisms and are able to manipulate the entire host immune response to avoid elimination [reviewed in Ref. (27)]. Accordingly, an alternative approach to tackle African trypanosomiasis is targeting the infection-associated immunopathology. For example, in HAT patients neurological complications are the major pathological feature, yet, an additional complication observed during the hemolymphatic stage is anemia (28, 29). In AAT, anemia is considered the most prominent immunopathological disease-related feature and the major cause of death due to Nagana (30). Importantly, in cattle, trypanotolerance has been referred to as the capacity of an animal to control severe anemia development which is assumed to be independent of parasitemia levels (21, 30). Moreover, Naessens et al. (31) showed using chimeric studies between trypanotolerant N’Dama (i.e., ancient cattle breeds/West African longhorn, *Bos taurus*) and trypanosusceptible Boran (more recently introduced cattle breeds, *Bos indicus*) that trypanotolerance is composed of two traits, (i) a better capacity to control parasitemia which is independent of the genetic origin of the hematopoietic tissue and (ii) a better ability to control anemia which is dependent on hematopoietic cells and thus a tolerant hematopoietic tissue genotype. Moreover, the capacity to control anemia is considered as the most important trait of the more resistant/trypanotolerant cattle (32). Yet, not only the genetic background (N’Dama versus Boran) but also other factors such as the age of the host, type of trypanosome spp. infecting, and nutrition can contribute to bovine trypanotolerance (33–38).

Tsetse fly mediated (i.e., natural infection mode) and experimental (i.e., using clonal parasites) murine models have been developed to allow a more detailed unraveling of the underlying mechanisms of trypanosomiasis-associated anemia development. Although most trypanosomes cannot be considered natural pathogens for rodents, experimental infections in mice may offer good models to identify the molecular pathways that mediate particular traits or pathological features such as anemia (39). Moreover, the genetic background of the mice was also found to contribute to susceptibility or tolerance as far as anemia is concerned, whereby during *T. brucei* and *T. congolense* infection C57BL/6 mice exhibited severe anemia (yet low parasitemia) while BALB/c mice exhibited greatly reduced anemia (yet higher parasitemia) (40, 41). However, there are some differences in the phenotype. Indeed, even the most tolerant mouse strains eventually succumb to the infection, while in the absence of other stress factors, tolerant cattle survive such challenge. So far, studies in murine models focusing mainly on “clonal or natural (tsetse transmitted)” *T. congolense* and *T. brucei* parasites have shown that similar as in the bovine system, chronic anemia does not seem to correlate with parasitemia or survival, but rather is a result of infection-elicited host responses, where B-cells do not seem to play a major role (40, 42). By contrast, cells of the mononuclear phagocyte system (MPS, i.e., tissue resident myeloid cells and inflammation-elicited/inflammatory myeloid cells derived from circulating monocytes) have been shown to play a key role in infection-associated pathogenicity/anemia development (43). Moreover, due to their sensing ability towards pathogen- and host-derived signals in the environment, their phagocytic capacity and functional plasticity in response to these signals, cells of the MPS are considered as a crucial immune population in both health and disease. A large number of studies, including our work, have begun to establish how the ontogeny/differentiation of these cells is tailored during the course of African trypanosome infections. In this review, we aim at (i) giving an overview of how trypanosome-derived and host-derived factors can affect the MPS and contribute to trypanosomosis-associated anemia development and (ii) discussing on potential intervention strategies to alleviate African trypanosomosis (AT)-associated anemia that might also have therapeutic potential.

## Anemia Development During African Trypanosome Infections

### Myeloid Cells As Key Players in the Parasite–Host Interaction and Trypanosomiasis-Associated Acute Anemia Development

**Figure 1:**
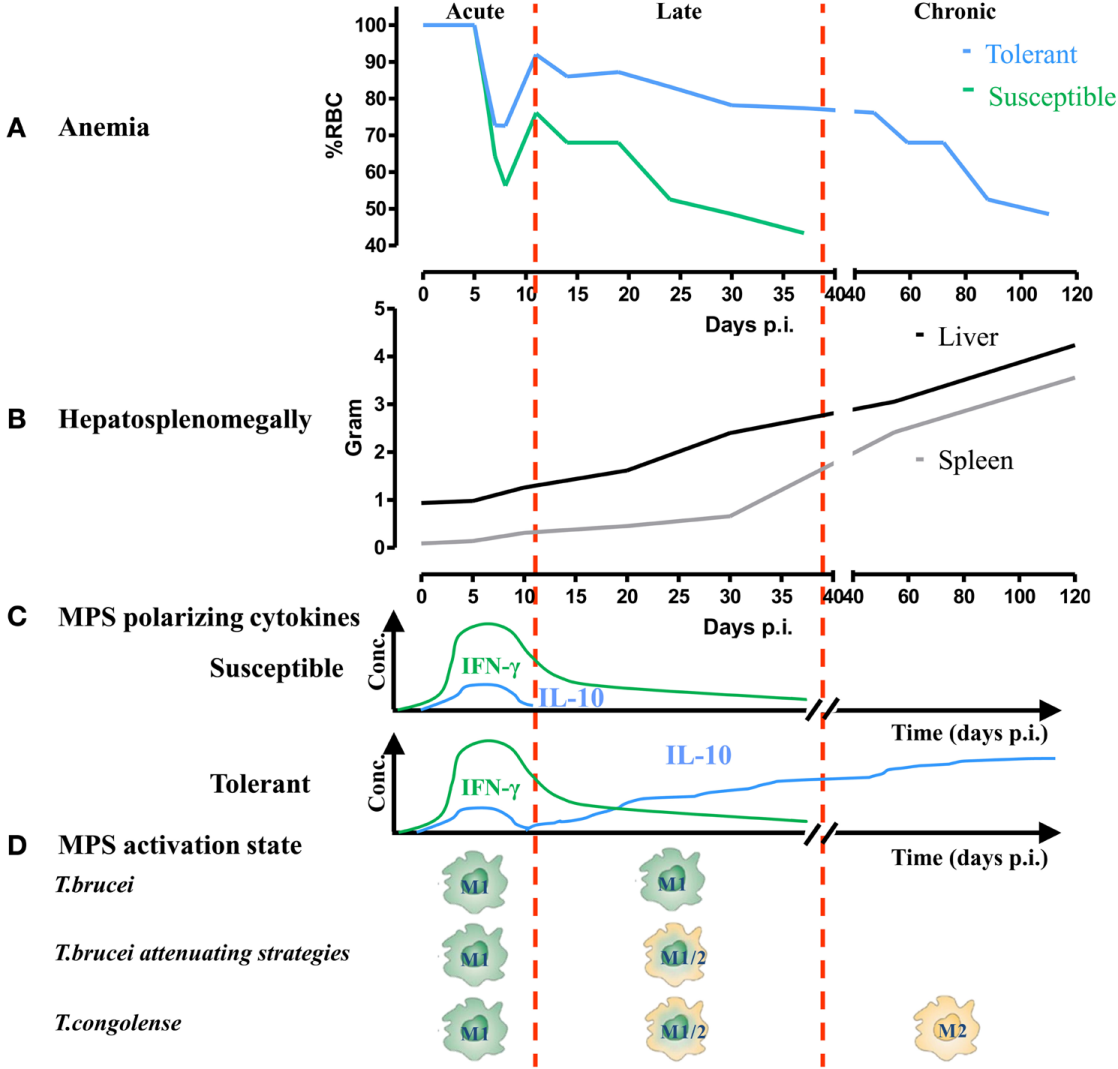
The activation state of myeloid cells correlates with anemia development during trypanosome infections in trypanosusceptible and trypanotolerant animals. **(A)** Anemia development in trypanosusceptible (green) and trypanotolerant (blue) animals during the course of infection. Anemia progression can be divided into (i) an acute phase characterized by a rapid drop in red blood cell (RBC) numbers (i.e., consumptive anemia) followed by a partial recovery phase, (ii) a late stage characterized in the susceptible model by a progressive decline in RBC numbers (below that of the acute phase) and host death. In the tolerant model, this decline is less pronounced and leads to (iii) a chronic phase (i.e., progressive anemia), whereby RBC numbers keep on declining till finally reaching levels of that of the acute phase. **(B)** Throughout the course of infection progressive hepatosplenomegaly occurs, whereby the onset of splenomegaly precedes hepatomegaly. At the late/chronic stage of infection, splenomegaly is more pronounced than hepatomegaly. **(C)** During the different stages of infection, the host produces different mononuclear phagocyte system (MPS) polarizing molecules. During the early/acute stage, both trypanosusceptible (upper, green) and trypanotolerant (lower, blue) animals produce IFN-γ (green line) required to trigger the induction of classically activated macrophages (M1), which is followed by a moderate induction of IL-10 (blue line) to dampen the pathogenic effects of the M1. Only in the trypanotolerant model, there is a second progressive increase in IL-10 during the late/chronic phase of infection, which is required to induce alternatively activated macrophages (M2). **(D)** Occurrence of M1 and M2 during the course of aggressive *Trypanosoma brucei* or *T. brucei* attenuating strategies (GPI-based strategy or AAV-10/anti-CD28) or less virulent *T. brucei* PLC^−/−^/*Trypanosoma congolense* infection.

The interaction between African trypanosomes and their mammalian host elicits the sequential activation of innate and adaptive immune responses. Being extracellular parasites, they are continuously confronted with the host’s immune system. However, through co-evolution, a well-balanced growth regulation system developed that allows sufficiently long parasite survival without killing its host to ensure transmission (44). This intricate balance consists of (i) a potent type 1 cellular/pro-inflammatory immune response and (ii) a strong humoral antiparasite B-cell response during the most prominent first peak parasitemia which collectively allows parasite control and temporary host resistance (42, 45). However, to avoid complete elimination, these extracellular parasites have developed various immune evasion mechanisms (consisting of antigenic variation, immunosuppression and B-cell depletion/loss of B-cell memory) to ensure progression/chronicity and transmission (46–50). Moreover, the early “beneficial” pro-inflammatory immune response mediated by the activated MPS, can culminate into severe collateral damage to the host if persistent. In this context, the level of the inflammatory immune response triggered and the capacity of the host to control this response determines whether immunopathology (i.e., anemia and tissue damage) develops and allows discriminating between trypanosusceptible and trypanotolerant animals (see Figure 1).

Experimental murine models, using gene-specific-deficient animals, have been very crucial in trying to unravel the mechanisms implicated in trypanosomiasis-associated pathogenicity and anemia in particular. In general, anemia occurs during all stages of a typical African trypanosome infection and can be divided into distinct phases (see Figure 1), (i) an early/acute stage whereby following/coinciding peak parasitemia clearance there is occurrence of a drastic drop in red blood cells (RBCs) numbers (i.e., acute or consumptive anemia) which is followed by a recovery phase and (ii) a more late/chronic phase coinciding with progressive anemia development. Accumulating evidence points to a pivotal role of myeloid cells in anemia development (see Figure 1). Hereby, their plasticity toward environmental triggers allows discriminating between classically activated macrophages (i.e., M1) and alternatively activated macrophages (i.e., M2). Moreover, the prevalence of M1 or M2 during the course of infection correlates with the severity of anemia (43). Consequently, both pro- and anti-inflammatory cytokines have been shown to be implicated in anemia onset and progression (51). In this section, different parasite- and host-derived factors contributing to both myeloid cell activation and to acute and chronic anemia development will be discussed. To this end, two different murine African trypanosome models, i.e., the *T. brucei* and *T. congolense* infection model, will be compared. It is important to mention that within the murine African trypanosomiasis model, *T. brucei* infections are associated with severe anemia (i.e., a more susceptible model) and *T. congolense* infections with reduced anemia (i.e., a more tolerant model). Emphasis will be put on the more thoroughly investigated murine *T. brucei* infection model. However, over the years more and more research has been conducted using the murine *T. congolense* infection model. Hence, we will also discuss, if possible, the common or distinct features underlying anemia development in both models.

#### Trypanosome-Derived Factors That Affect Myeloid Cell Activation during the Early/Acute Stage of Trypanosome Infection

**Figure 2:**
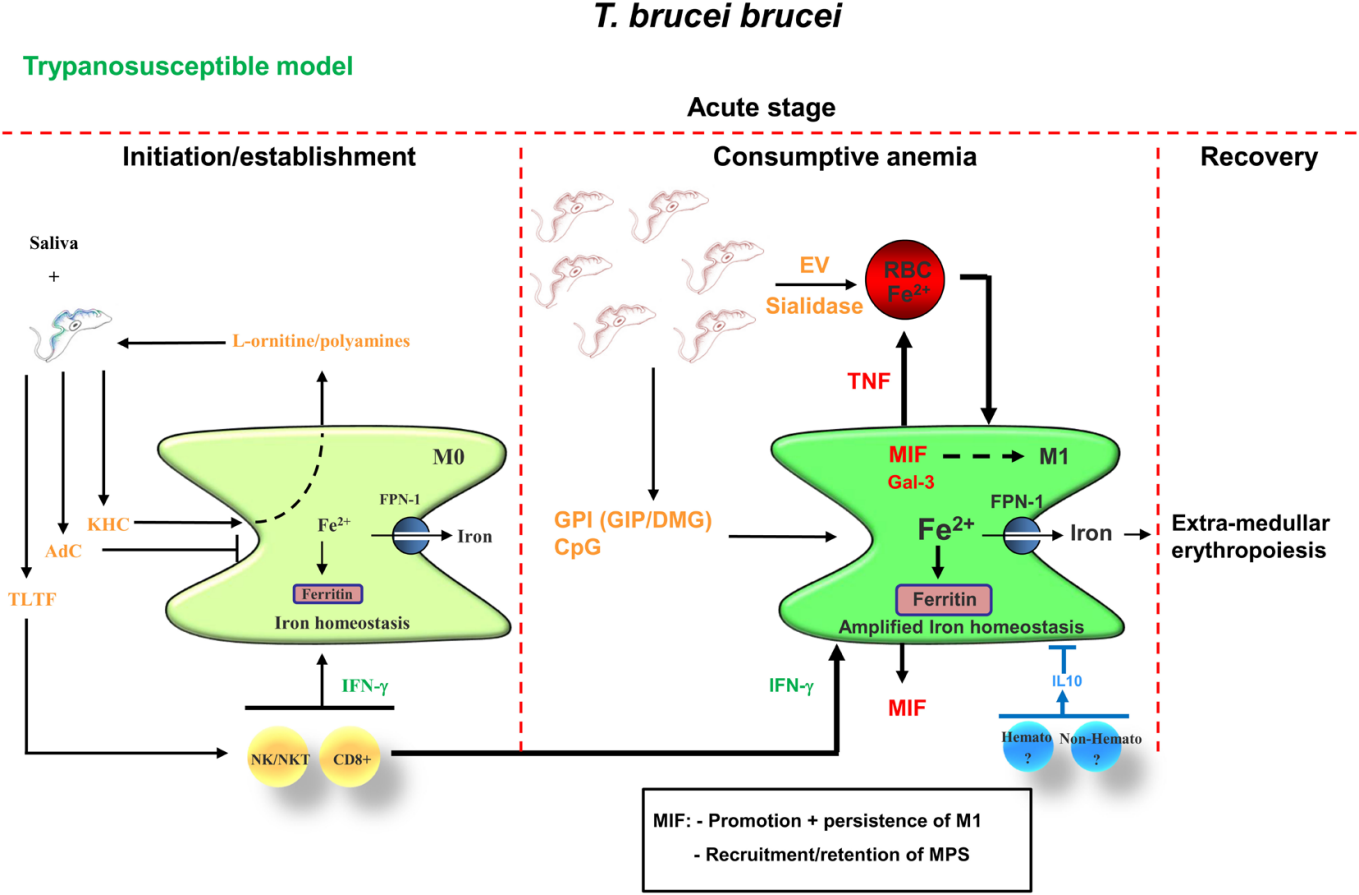
Proposed model for acute anemia development during trypanosome infections in trypanosusceptible animals (i.e., *Trypanosoma brucei*). During the early stage of infection, saliva components in concert with parasite-derived factors such as KHC [fueling parasite nutrient production (i.e., polyamines)] and AdC (dampening the potential of M1), temporarily disable/attenuate the M1-mediated innate immune response, thereby allowing parasite initiation/establishment. In addition, parasites release TLTF to trigger IFN-γ production by NK/NKT and CD8^+^ T cells, which will promote M1 cell activation. At the peak of parasitemia, these IFN-γ-primed M1 in concert with parasite-derived factors such as GPI-VSG (GIP/DMG) and CpG fuel M1 to produce pro-inflammatory molecules such as TNF, MIF, and Gal-3. In turn, TNF in concert with parasite-released extracellular vesicles (EVs) and/or sialidase trigger/enhance RBC senescence and hence erythrophagocytosis by M1 cells, leading to the development of acute anemia (i.e., hemophagocytic/consumptive anemia). Both MIF and Gal-3 promote M1 polarization and at this stage, there is an amplification of the iron homeostasis [i.e., increased iron storage (ferritin) and export (FPN-1)], which triggers extramedullary erythropoiesis. MIF also promotes recruitment/retention of mononuclear phagocytes and neutrophils, which in turn fuel the pathogenic effects of M1. At this stage, the host transiently produces IL-10, which is required to dampen the pathogenic effects of the M1 and in concert with the extramedullary erythropoiesis results in a partial recovery from acute anemia. Abbreviations: TLTF, T-lymphocyte triggering factor; KHC, kinesin heavy chain; AdC, adenylate cyclase; GPI, glycosylphosphatidylinositol; GIP, glycosylinosytolphosphate; RBC, red blood cell; MIF, macrophage migration inhibitory factor; Gal-3, galectin-3; FPN-1, ferroportin-1; Hemato, hematopoietic; Non-Hemato, non-hematopoietic; DMG, dimyristoylglycerol; VSG, variant surface glycoprotein.

Upon the bite of a trypanosome-infected tsetse fly, metacyclic parasites expressing a heterologous variant surface glycoprotein (VSG) coat (i.e., metacyclic VSG) that prevents early detection/elimination (52), are inoculated. Already during the early stage of infection, trypanosomes release factors that alone or in concert with saliva components can dampen/impair the activation of the host’s immune response, to generate a privileged “micro”-environment to allow infection establishment [reviewed in Ref. (27) and shown in Figure 2, left panel]. Of note, with respect to parasite-released factors that could modulate the host MPS, most research so far has been performed using the model parasite *T. brucei* and remain to be determined for the *T. congolense* model. For instance, studies using the *T. brucei* model parasite revealed that they harbor a kinesin heavy chain 1 (TbKHC1), which induces IL-10 and arginase-1, signals through SIGN-R1 in myeloid cells and downregulates inducible nitric oxide synthase activity (53). In turn, this stimulates the production by the host of l-ornithine and hereby the synthesis of polyamines, which promotes early parasite growth (54). Consequently, IL-10/arginase-1-producing immune cells are impaired in their capacity to destroy the parasite, favoring parasite settlement. Another factor trypanosomes use to establish infection is the *T. brucei* adenylate cyclase, which converts ATP into cyclic adenosine monophosphate (cAMP) and is upregulated upon phagocytosis by M1 cells (55). This phenomenon leads to the inhibition/suppression of macrophage activation and consequently to an impaired production of parasite controlling molecules (56–59). Hence, it seems that trypanosomes have developed a system, where altruistic phagocytosed parasites can “temporarily” tempering/disabling the M1-mediated innate immune response required for parasite control (see Figure 2). In turn, this favors the induction of M2 and paves the way for initiation and establishment of the first wave of parasitemia.

Following initial infection, trypanosomes also “deliberately” trigger in a well-timed manner host cellular responses, whereby myeloid cells get activated *via* the combined exposure of (i) parasite-released components (i.e., pathogen-associated molecular patterns) such as the soluble and membrane-bound/glycosylphosphatidylinositol (GPI)-anchored VSG (sVSG and mfVSG, respectively) and CpG-DNA and (ii) NK/NKT/T-cell released IFN-γ, which most likely is mediated *via* a trypanosome-lymphocyte-triggering-factor (TLTF) (see Figure 2, right panel) (45, 60–64). This combination triggers the activation of M1 cells, which in turn release pro-inflammatory molecules such as tumor necrosis factor (TNF-α) and nitric oxide (NO). However, the timing of exposure of these (parasite- and host-derived) components is pivotal in the development of the immune response toward the parasites. These key events controlling host resistance occur within a short time period following initial exposure to the parasite-derived components. Indeed, trypanosomes can cleave their GPI-anchored VSG molecules from the membrane by the trypanosome GPI-phospholipase-C, which results in the release of soluble glycosylinositolphosphate VSG (GIP-sVSG) and the retention of the dimyristoylglycerol (DMG) moiety in the parasites’ membrane (65–68). Both components (DMG and GIP-VSG) exhibit a distinct macrophage-activating potential (60, 62). For example, the GIP-VSG moiety is recognized by a type A scavenger receptor (SR-A) expressed mainly on mononuclear cells (e.g., macrophages and dendritic cells) leading to the concentration-dependent activation of NF-κB and MAPK pathways and expression of pro-inflammatory genes such as (TNF-α, IL-6, IL12p40, and granulocyte-macrophage colony-stimulating factor) in a MyD88 dependent manner (61, 63). However, the fluctuating levels of parasite (e.g., GIP-sVSG) and host (e.g., IFN-γ) factors during infection act to control macrophage activity in a complex and subtle way, with the outcome determined by the concentration of each mediator, the sequential pattern of its production, and the microenvironment of the target macrophage (69). For instance, during the early/initial stage of infection, the GIP-sVSG released before a high level of IFN-γ production prevents a prominent strong pro-inflammatory immune response and hence favors parasite establishment. Yet, if the IFN-γ levels increase this will prime macrophages to respond stronger toward the parasite-derived GIP-sVSG, which in turn will fuel M1 cells to mount a prominent pro-inflammatory immune response.

#### Host-Derived Factors That Affect Myeloid Cell Activation during the Early/Acute Stage of Trypanosome Infection

Using gene-deficient mice or neutralizing antibodies it was shown that the sequential production of IFN-γ by NK, NKT, as well as CD8^+^ and CD4^+^ T cells during the early stage of trypanosome infection seems to be crucial to initiate acute inflammation-associated anemia (70), also termed consumptive anemia (71, 72) (see Figure 2, left panel). In this scenario, IFN-γ activates M1 cells, which in turn allows parasite elimination/removal, but at the same time also promotes the M1-mediated enhanced uptake of RBCs resulting in a first rapid drop in RBC numbers. Indeed, IFN-γ receptor-deficient mice were found to exhibit greatly reduced acute anemia levels (73), coinciding with a reduced influx of myeloid-derived cells, e.g., neutrophils and M1, within the liver that exhibit an impaired erythrophagocytosis capacity (70). Increased levels of host-derived IFN-γ furthermore induce splenomegaly (71), which is typically observed during the acute stage. Recently, Stijlemans et al. (74) demonstrated using a pHrodo-based assay that during the early stage of *T. brucei* infection, CD11b^+^Ly6G^+^ neutrophils, CD11b^+^Ly6C^high^ monocytic cells, as well as splenic CD11b^+^F4/80^+^ myeloid cells exhibit an enhanced erythrophagocytosis capacity that might account for the occurrence of severe acute-stage non-hemolytic anemia. Interestingly, it was shown by others that enhanced erythrophagocytosis is associated with the mobilization of Ly6C^high^ monocytes in a CCR2-dependent manner from the bone marrow into the blood (75), which accumulate mainly within the liver and subsequently ingest stressed/senescent erythrocytes. These cells differentiate into iron-recycling/ferroportin-1 (FPN-1, sole iron exporting molecule)-expressing tissue macrophages and subsequently into iron-recycling Kupffer-like cells, which is a natural mechanism to preserve homeostasis during fluctuations of erythrocyte integrity (76).

#### Host- and Parasite-Derived Factors That Contribute to Acute Anemia

It was shown that RBCs from infected (i.e., day 6 postinfection) wild-type (WT) mice exhibited an enhanced osmotic fragility and an altered fatty acid membrane composition compared with RBCs from non-infected WT mice (70). This change in RBC fragility was not due to IFN-γ but might be due to host-derived factors such as TNF-α produced by M1 cells (77–80). Indeed, TNF-α could be a driving force for the observed changes in RBC fragility given that it was shown that it can decrease the RBC half-life and thereby fuel RBC senescence/elimination (81). The importance of host-derived factors such as TNF-α in acute anemia development was further substantiated by the observation that *T. brucei*-infected TNF-α-deficient (TNF-α^−/−^) mice exhibited greatly reduced acute anemia levels compared with control WT mice (see Table 1). Thereafter, RBC levels in TNF-α^−/−^ mice remained elevated. By contrast, in the *T. congolense* model, TNF-α^−/−^ mice exhibited similar acute anemia (and chronic) levels as control WT mice (82, 83), suggesting that in this model the underlying mechanisms of anemia development are different. Besides TNF-α produced by activated myeloid cells, NO was also found to be an important factor affecting *T. brucei*-associated acute anemia development. Indeed, treating C57BL/6 mice with l-NAME (a typical inhibitor of NO synthase) alleviated acute anemia development (coinciding with reduced peak parasitemia) and was proposed to affect proliferation of immature erythrocytes or hematopoietic stem cells (73, 84). In line with these observations, mice treated with corticosteroids (which downregulates NO synthesis) exhibited an alleviated anemia development (85). However, more research is required to unravel at which level NO affects *T. brucei*-associated acute anemia development.

**Table 1:** Overview of acute and chronic anemia development in different *Trypanosoma brucei*-infected mouse strains.

Mouse model	Acute anemia	Late/chronic anemia	Reference
BALB/c	+	+	(41, 42)
C57BL/6 (WT)	+++	+++	(41, 42, 74, 83, 90)
^a^B-cell^−/−^	+++	+++	(42)
^a^Nu/Nu	+	+	(70)
^a^IFN-γ^−/−^ or IFN-γR^−/−^	+	+	(70, 73)
^a^CD8^−/−^	+	++	(70)
^a^CD4^−/−^	+++	+ to ++	(70)
C57BL/6 + anti-NK1.1	+	+++	(70)
C57BL/6 + anti-CD28 superagonist	+	+++	(130)
C57BL/6 + AAV-IL-10	ND	+	(131)
C57BL/6 + GPI-based strategy	+	+	(95, 132)
^a^IL-10^−/−^	+++	ND	(131)
^a^TNF^−/−^	+	+	(59, 83, 118)
^a^TNF-R1^−/−^	+++	+++	(41)
^a^TNF-R2^−/−^	++	+	(41)
^a^LT-α^−/−^	++	+ to +++	(118)
C57BL/6 + L-NAME	+	ND	(84)
^a^MIF^−/−^	+	+	(93)
^a^Gal-3^−/−^	+	+	(133)

*+, Mild anemia (<25% drop in RBCs); ++, moderate anemia (25–35% drop in RBCs); +++, severe anemia (>35% drop in RBCs); ND, not determined; RBC, red blood cell; AAV-IL-10, alternatively, adenoviral delivery of IL-10; Gal-3, galectin-3; MIF, macrophage migration inhibitory factor; WT, wild-type; LT-α, lymphotoxin-alpha*.

*^a^Gene-deficient mice in C57BL/6 background*.

Also parasite-derived factors such as sialidases in the case of *T. congolense* or extracellular vesicles (EVs) in the case of *T. brucei* infections could contribute to modifications of RBCs and thereby promote elimination (77–80). Indeed, it was proposed at least for the murine *T. brucei* model that during the acute stage, trypanosomes release EVs (filled with intracellular parasite cargo as well as VSG) that can fuse with RBCs. This causes a change in the physical properties of the RBC membrane, which enhances erythrophagocytosis and thereby fuels anemia development. In this context, it could be that binding of mfVSG (present in the EVs) to the RBC surface sensitizes erythrocytes to anti-VSG antibody-mediated complement lysis (86). In addition, this observation might also explain how active adenylate cyclase, playing a key role in increasing cAMP in host cells resulting in the activation of protein kinase A and downregulation of TNF-α, could be transferred from the parasite to the mammalian host (see above). Indeed, the highly fusogenic EVs containing this enzyme might be transferred to recipient host cells, thereby increasing the intracellular levels of cAMP. Given that these EVs are mainly produced at the peak of parasitemia, they might in one way stimulate RBC elimination and at the same time dampen subsequent inflammatory reactions, thereby allowing the next wave of parasites to escape. Also for the murine and bovine *T. congolense* model, factors such as congopain and sialidases were suggested to contribute directly/indirectly to anemia development, by damaging RBCs that results in the exposure of erythrophagocytosis promoting targets (phosphatidylserine) on the RBC membrane (78, 87, 88).

Different factors might account for the occurrence of acute anemia in both the *T. brucei* as well as the *T. congolense* murine infection model. Hence, the acute stage of anemia could be due to a “natural” reaction of the host following infection as well as to parasite-derived factors resulting in a rapid drop in RBC numbers due to enhanced erythrophagocytosis (89). At this stage of infection, due to the enhance erythrophagocytosis, there is an amplification of the iron-homeostasis metabolism (90), resulting in an increased release of iron to fuel the enhanced demand for erythropoiesis (Figure 2, right panel).

#### Transition from Acute to Chronic Anemia: The Recovery Phase

Following this acute anemia phase, there is a transient recovery phase in both the *T. brucei* as well as the *T. congolense* infection model (see Figure 1), as a natural response of the host to control/alleviate acute anemia development. Of note, within the *T. brucei* infection model, this recovery was more pronounced in the IFN-γR^−/−^, CD8^−/−^, TNF-α^−/−^, and TNF-R2^−/−^ mice, suggesting that a reduced early pro-inflammatory response/insult allows better recovery from acute anemia. However, so far, the exact mechanism(s) involved are not well characterized. From other experimental models of acute anemia (phenylhydrazine-induced injection or bleeding), it could be inferred that this is most likely due to an enhanced extramedullary erythropoiesis occurring mainly in the spleen and to a lesser extent in the liver and coincides with the occurrence of hepatosplenomegaly. In this context, both in the *T. brucei* and *T. congolense* infection model, hepatosplenomegaly has been documented starting already during the early stages of infection, and coincided with an increase in immature RBC numbers within the splenic compartment (91–95). It is generally known that anemia induces tissue hypoxia, which in turn triggers the activation of a physiological stress response (i.e., stress erythropoiesis) designed to increase oxygen delivery to tissues by rapidly generating large numbers of erythrocytes (96). Moreover, tissue hypoxia triggers the induction of erythropoietin (EPO) in the kidney (97), which drives the expansion and differentiation of erythroid progenitors. Of note, during murine and bovine trypanosome infections, serum EPO levels are increased during both the acute and chronic stage of infection (83, 98). Subsequently, the bone marrow progenitor cells migrating into the spleen or stress erythroid progenitors resident in the spleen expand and differentiate in response to bone morphogenetic protein 4 (BMP4) and Hedgehog, which act in concert with signals previously associated with stress erythropoiesis, such as EPO, stem cell factor and hypoxia, to replenish the pool of stress erythroid progenitors (96, 99, 100). Whether macrophages play also a role at the level of erythropoiesis within the African trypanosome model remains to be further investigated. However, it was shown that upon anemia or stress, macrophage-dependent erythropoiesis (within erythroblastic islands) is needed to adequately respond to produce enough erythrocytes to alleviate the shortage (101, 102). Interestingly, in experimental *T. congolense* infections in rats, erythroblastic islands were found to expand already during the early stages of infection within the bone marrow (103). It is important to mention that following this prominent pro-inflammatory immune response and coinciding with the partial recovery of acute anemia, the host is able to trigger a “transient” anti-inflammatory immune response, whereby IL-10 was shown to play a key role (104, 105). At this stage, CD4^+^ T cells were shown to be important IL-10-producing cells to dampen the pathogenic effects of the IFN-γ-induced M1 (106). Yet, it can not be excluded that other cells (hematopoietic or non-hematopoietic) might also contribute (see Figure 2, right panel). Recently, it was also suggested that IL-27 can play a key role in dampening the pathogenic effects of T cell-mediated IFN-γ during *T. brucei* and *T. congolense* infection without affecting IL-10 levels (107).

### Myeloid Cells As Key Players during the Late/Chronic/Progressive Stage of Trypanosomiasis-Associated Anemia Development

**Figure 3:**
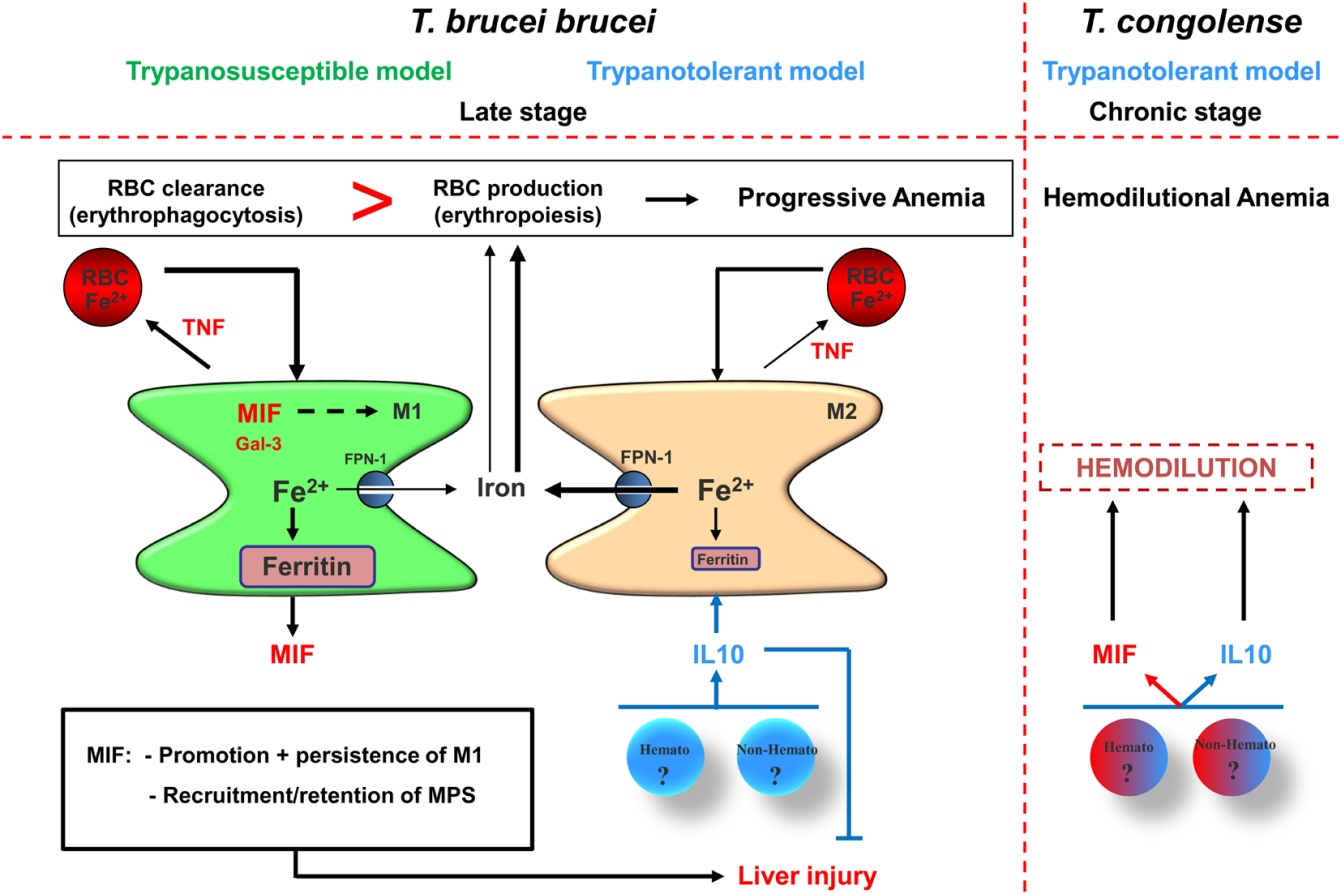
Proposed model for pathogenicity development during the late/chronic stage of infection in trypanosusceptible and trypanotolerant animals. (Left panel) Following recovery from acute anemia (see Figure 2), trypanosusceptible animals are unable to trigger a second wave of IL-10 production resulting in persistence of M1, which is most likely promoted *via* macrophage migration inhibitory factor (MIF) and galectin-3. These M1 produce TNF that further promotes erythrophagocytosis and exhibit iron accumulation [increased ferritin and reduced ferroportin-1 (FPN-1) expression], resulting in iron deprivation from erythropoiesis and collectively leading to progressive anemia development. MIF also promotes myeloid cell recruitment/retention leading to liver injury. By contrast, in trypanotolerant animals, following the acute stage of infection, hematopoietic (hemato) or non-hematopoietic (non-hemato) cells produce IL-10, which dampens the pathogenic effects of the M1 by triggering the induction of M2. These M2 exhibit reduced erythrophagocytosis and reduced iron retention, leading to a more efficient erythropoiesis and in turn to reduced progressive anemia development. IL-10 also reduces liver injury by dampening the pathogenic effects of the M1. (Right panel) However, the chronic production of MIF by M1 and persistent IL-10 production can collectively promote progressive hemodilution, which is most likely due to enhanced (hepato)-splenomegaly. In turn, the hemodilution will cause an apparent anemia development in trypanotolerant animals, resulting in thrombocytopenia and impaired coagulation in infected mice. The heme catabolism following the chronically induced erythrophagocytosis leads to iron accumulation in myeloid cells and to hyperbilirubinemia. The combination of liver injury and hemodilution leads to declined serum albumin levels, thereby preventing efficient removal of toxic molecules from the circulation, including bilirubin, and cause multiple organ failure that culminates in reduced survival of the infected host.

Following partial recovery from acute anemia, there is a new equilibrium established, which is different from the steady-state situation (see Figure 1). At this stage, the capacity of the host to keep the balance between erythrophagocytosis and erythropoiesis determines whether anemia persists. This also allows discriminating between susceptible and tolerant animals as far as anemia is concerned, whereby the activation stage of the myeloid cells determines the degree of anemia. Indeed, on one hand, trypanosusceptible animals maintain a prominent/polarized M1 activation state and exhibit progressive anemia (i.e., *T. b. brucei* model), which resembles anemia of chronic disease or anemia of inflammation (90). This is characterized by an enhanced erythrophagocytosis and impaired/reduced erythropoiesis that is linked to a perturbed iron homeostasis including altered iron recycling by macrophages and iron sequestration (Figure 2). Therefore, the iron-processing pathway is skewed toward iron sequestration (40, 90), as evidenced by increased ferritin expression (main iron storage molecule) and reduced FPN-1 (sole iron exporter), while enhanced uptake of RBC/iron-containing compounds is maintained (see Figure 3, left panel). Moreover, iron sequestration by cells of the MPS can fuel their M1-type activation status and limit iron availability for erythropoiesis (108–111), thereby contributing to the persistence of anemia.

In this context, it was shown that pro-inflammatory cytokines such as IFN-γ, TNF, IL-1, and IL-6 can affect iron-homeostasis regulation as well as erythropoiesis. Indeed, during homeostasis, there is a balance between RBC destruction and production, where the iron availability is adequate to accommodate the host’s erythropoietic demand (112). Yet, during inflammation this balance is shifted toward an enhanced RBC destruction and impaired/insufficient production or RBCs, leading to anemia. These pro-inflammatory cytokines trigger (i) the upregulation of the divalent metal transporter-1, which increases iron uptake by the reticuloendothelial cells, (ii) an enhanced ferritin expression (i.e., iron storage molecule), and (iii) a downregulation of FPN-1 expression thereby promoting iron retention within the MPS (113, 114). This will cause a deprivation of iron from erythropoiesis. At the same time, these pro-inflammatory cytokines inhibit erythropoiesis by (i) downregulating EPO receptors thereby impairing the EPO-mediated effects, (ii) increasing erythroid apoptosis, and (iii) antagonizing pro-hematopoietic factors (115, 116).

On the other hand, trypanotolerant animals are able to switch to a protective anti-inflammatory response (induced *via* IL-10), which is reflected by the occurrence of M2 cells that in concert with IL-10 are able to dampen the pathological effects of the M1 cells, and exhibit an alleviated anemia development [i.e., *T. brucei* attenuation strategies, phospholipase-C-deficient (*PLC*^−/−^) *T. brucei* and *T. congolense* model]. Moreover, trypanotolerant animals in contrast to trypanosusceptible animals exhibit a restored iron homeostasis (i.e., an enhanced FPN-1 and reduced ferritin expression) and increased iron availability for erythropoiesis (95). In addition, M2 cells exhibit a reduced erythrophagocytosis capacity, whereby iron homeostasis is skewed toward export (117). It is important to mention that in both the *T. brucei* and *T. congolense* infection model, the host is able to produce IL-10 during the acute stage of anemia to dampen the pathogenic effects mediated *via* the pronounced pro-inflammatory response, which is linked to the first wave of parasitemia control. However, it seems that in the *T. brucei* (susceptible) model the host is unable to retrigger IL-10 induction to dampen the second wave of inflammation, while in the *PLC^−/−^T. brucei*/*T. congolense* (tolerant) model the host is able to mount a second progressive IL-10 response, which is sufficient to dampen the lower level of inflammation. This difference in ability to trigger a second wave of IL-10 (or alternatively, maintain an IL-10 triggering potential) is also reflected at the level of differences in M1 and M2 between susceptible and tolerant animals.

#### Myeloid Cell Activation in the *T. brucei* (Susceptible) versus *T. congolense* (Tolerant) Model

As far as the *T. brucei* model (using AnTat1.1E) is concerned, there is a persistent M1 activation contributing to severe anemia and tissue injury (see Figures 1 and 3). It was shown that the TNF-family members [TNF-α and lymphotoxin-alpha (LT-α)] play a key role in chronic/progressive anemia development by signaling *via* their dedicated receptors [TNF-R1 or p55 (CD120a), TNF-R2, or p75 (CD120b)] (see Table 1) (118). Thus, TNF-α-deficient (TNF-α^−/−^) or TNF-R2-deficient (TNF-R2^−/−^) mice exhibited greatly reduced chronic anemia compared with WT or TNF-R1-deficient (TNF-R1^−/−^) mice (41, 59), suggesting that TNF-R2 signaling mediates infection-associated pathology, whereas TNF-R1 signaling has little or no impact on the *T. brucei* infection. Moreover, the serum levels of soluble TNF-R2 after shedding, which impaired TNF-α-signaling pathways in myeloid cells, correlated with the inhibition of TNF-mediated immunopathology. Moreover, the low ratio of total TNF-α to soluble TNF-R2 observed in BALB/c mice may account for the lack of TNF-mediated pathology, whereas an increased ratio in C57BL/6 mice coincided with the severe pathology/anemia. Using LT-α^−/−^ mice, it was shown that the TIP sequence (i.e., lectin-like domain) of TNF-α does not seem to play a role in anemia development (118). Importantly, TNF-α and LT-α have high amino acid sequence homology and both bind to the TNF-α p55 and p75 receptors (TNF-R1 and -R2, respectively) as soluble homotrimers (119), yet they exhibit alterations in the TIP sequences (120). For example, TNF-α exerts a lectin-like affinity for several carbohydrate sequences while LT-α does not (121). These LT-α^−/−^ mice were shown to exhibit during the middle stage of infection (days 10–28) a greatly reduced anemia compared with WT mice, which coincided with reduced TNF-α induction in LT-α^−/−^ mice during this stage. However, during the final stage of infection, serum TNF-α reaches the same levels in both LT-α^−/−^ and WT mice, concomitant with similar anemia levels in both mice groups. A possible explanation for this might be that TNF-α is also an important negative regulator of erythropoiesis and this aspect might predominate at later stages of infection (51, 122). Hence, strategies to reduce TNF signaling or allowing switching from M1 toward M2 might also be valuable to alleviate chronic anemia development. Different factors were found to contribute to the ability of the host to switch from M1 to M2 and the ability to maintain/trigger IL-10 during the later stages was shown to be detrimental to attenuate anemia.

Collectively, it seems that the mechanisms underlying trypanosomiasis-associated anemia are multifactorial and the relative contribution of each mechanism will differ according to the host–parasite model, the phase of anemia development and the severity of infection and is probably caused by massive extravascular erythrophagocytosis by an expanded MPS in concert with an inadequate erythropoiesis.

## Potential Intervention Strategies to Alleviate At-Associated Anemia

### The Parasite Strain Used Determines the MPS Activation State and Anemia Development

Typically, in the experimental *T. brucei* C57BL/6 model, the myeloid cells are polarized into an M1 state, which is promoted due to the inability of the host to sustain a strong anti-inflammatory immune response. By contrast, in the less aggressive model experimental *PLC^−/−^T. brucei* C57BL/6 model, there is a switch from M1 toward M2 mediated *via* IL-10, coinciding with reduced pathology (anemia/tissue injury) and prolonged survival. A possible explanation for this switch toward M2 in the *PLC^−/−^T. brucei* model and not in the WT *T. brucei* model might rely in the fact that the PLC is required to sustain M1 by (i) allowing the release of GPI-anchored proteins (encompassing the GIP) to stimulate macrophages to secrete pro-inflammatory molecules (TNF-α, IL-1, IL-6, and NO) and/or (ii) trigger CD1d-restricted NKT cells to secrete IFN-γ thereby triggering a very strong type 1 immune response (60, 123). Indeed, it was shown that in the *PLC^−/−^T. brucei* C57BL/6 model the lower parasitemia coincided with reduced early IFN-γ production and subsequent attenuated MPS-derived pro-inflammatory cytokine production, reflecting a reduced type 1 immune response mounted (124–126). The crucial role of IFN-γ and IL-10 during infection was further substantiated using gene-deficient mice, where the absence of IL-10 coincided with high pathology and early mortality. Although the source of IL-10 was not thoroughly investigated within the *PLC^−/−^T. brucei* model, some data suggest the involvement of CD4^+^ T cells (124). Interestingly, infections of *PLC^−/−^T. brucei* parasites using C57BL/6 x BALB/c (B6B-F1) mice were found to exhibit striking similarities with that of the trypanotolerant N’Dama cattle naturally infected with *T. congolense*. These latter include (i) lower parasitemia, (ii) prolonged survival, (iii) increased type II and decreased type I immune responses, and (iv) reduced pathology and minimal clinical symptoms during the course of infection (127, 128). Therefore, *PLC^−/−^T. brucei*-infected B6B-F1 mice represent a suitable model to study the immune responses during bovine *T. congolense* infections. Within the *T. congolense* model in C57BL/6 mice, it was shown that spleen and liver regulatory T cells (Foxp3^+^ Tregs) were an important source of IL-10, thereby limiting the production of early IFN-γ by T cells and in that way lowering pathology. Besides Tregs, also myeloid-derived IL-10 was shown to play an important role in limiting the production of pathogenic TNF-α by M1 cells (characterized as CD11b^+^Ly6C^+^) through induction of nuclear translocation of the NF-κB p50 member (129). However, it cannot be excluded that other hematopoietic and non-hematopoietic cells can be potential sources of IL-10 during the course of *T. congolense* infection (Figure 3).

### IL-10-Inducing Strategies to Modulate the MPS Activation State and Anemia Development

As mentioned before, the capacity of the host to induce IL-10 immediately after the induction of a prominent pro-inflammatory immune response mediated *via* M1 cells determines whether pathology/anemia develops/is alleviated or not. This opens perspectives for potential IL-10 triggering/promoting intervention strategies aiming at triggering an M1 toward M2 switch, thereby reducing pathology development. So far, several strategies have been used to demonstrate/strengthen the pivotal role of IL-10 in reducing trypanosomiasis-associated pathogenicity using the susceptible *T. brucei* model. For instance, transient anti-CD28 superagonist antibody treatment (inducing regulatory T cells and M2) in the *T. brucei* model attenuated acute anemia development (130). Given that this treatment was not continued during the chronic phase of infection its effects during this stage remain to be determined. Alternatively, adenoviral delivery of IL-10 in the *T. brucei* model coincided with an alleviated pathology/anemia development (131), during the chronic phase of infection (see Table 1). Also a GPI-based treatment strategy, where the parasite-derived GPI moiety (i.e., most potent parasite-derived TNF-inducing molecule involved in M1 triggering) was used to reprogram macrophages toward an anti-inflammatory state (i.e., reflected by a reduced inflammatory cytokine production and increased IL-10 production), was shown to alleviate anemia in both clonal as well as natural/non-clonal *T. brucei* infections (132). This strategy allowed reducing RBC destruction, normalizing iron homeostasis (i.e., a shift in increased liver expression of iron storage toward iron export genes) and restoring erythropoiesis [i.e., increased erythropoiesis in the bone marrow and extramedullary sites (spleen)] (95). Interestingly, this GPI-based treatment also alleviated “chronic” anemia development during experimental *T. congolense* as well as *T. evansi* infections suggesting a wide applicability.

### M1-Promoting Factors Are Prime Targets to Attenuate Anemia

Given that M1 cells are major contributors to anemia development, identification of M1-derived pathological factors might open perspectives to attenuate the pathology. An approach to identify potential M1-derived pathology inducing/promoting factors consisted of scrutinizing a GPI-based strategy, which enabled a straightforward comparison between trypanotolerance and trypanosusceptibility in *T. brucei*-infected C57BL/6 mice, independent of the genetic background of the host (95). A resulting comparative gene expression analysis of M1-polarizing molecules and/or molecules involved in enhancing erythrophagocytosis identified galectin-3 (Gal-3) and macrophage migration inhibitory factor (MIF) as potential candidates. Both molecules were indeed found to contribute to anemia development during *T. brucei* infections by affecting/regulating different aspects of the host’s immune response. As far as Gal-3 (i.e., a family member of beta-galactoside-binding animal lectins) is concerned, it was shown that *Gal-3^−/−^* mice manifested higher IL-10 levels that can exert an influence on iron uptake and counteract the effects of IFN-γ (133). Hence, Gal-3 can promote persistence of M1 and regulate the expression of iron-homeostasis genes, favoring iron storage, which ultimately culminates in iron shortage for erythropoiesis and exacerbate inflammation-associated anemia development (Figures 2 and 3) (133, 134). In addition, given the negative effect of Gal-3 on the induction of IL-10, a persistent inflammatory response is ensured in presence of Gal-3. As far as MIF is concerned, this “early response” cytokine is expressed by numerous cell types, including myeloid cells, plays a key role in innate and adaptive immunity and was shown to be involved in many protozoan infections (135–137). Using *Mif^−/−^* mice it was shown that this upstream regulator of the inflammatory cascade contributed to inflammation-associated pathogenicity by (i) sustaining a persistent pro-inflammatory type I immune response (impairing IL-10 production) and (ii) maintaining/enhancing the recruitment of pathogenic monocytic cells and neutrophils in the liver whereby neutrophil-derived MIF contributed significantly to enhanced TNF production and liver damage (Figures 2 and 3) (93). The pivotal role of MIF within the African trypanosomiasis model regarding the persistence of inflammation might be multifactorial. For instance, endogenous MIF has been shown to (i) promote macrophage-mediated inflammatory responses *via* induction of CC chemokine ligand 2 expression, thereby promoting the recruitment of monocytes into affected areas and (ii) exert a regulatory role in cellular responsiveness to key pro-inflammatory cytokines TNF and IL-1 *via* upregulation of cytokine receptor-dependent MAPK signaling (i.e., upregulation of TNF-R1 and IL-1R expression, respectively) independent of NF-κB (138, 139). Hence, by both attracting and activating monocyte/macrophages, MIF may contribute to the initiation and perpetuation of detrimental inflammation associated with diseases such as African trypanosomiasis. In addition, MIF importantly contributed to anemia development by (i) promoting iron accumulation in liver myeloid cells, (ii) enhancing RBC clearance, and (iii) suppressing erythropoiesis at later stages of erythroblast differentiation (Figure 3) (93). Interestingly, MIF was also shown to be a potential pathogenic molecule playing a key role in chronic anemia development during *T. congolense* infections by (i) promoting erythrophagocytosis, (ii) blocking extramedullary erythropoiesis and RBC maturation, and (iii) triggering hemodilution (Figure 3) (94).

Overall, it seems that during murine *T. brucei* and *T. congolense* infections anemia is mainly due to enhanced erythrophagocytosis combined with enhanced but inadequate extramedullary erythropoiesis. Yet, during *T. congolense* but not *T. brucei* infections, hemodilution (involving massive hepatosplenomegaly) seems to be an additional factor contributing to chronic anemia development (93, 94). However, it might be that within the *T. brucei* model the contribution of hepatosplenomegaly to hemodilution is minor or not reached within this “short” time period. This notion is strengthened by the fact that there was no correlation between anemia and hemodilution (involving hepatosplenomegaly) in *T. brucei* and *T. congolense*-infected rats within the same time window (92). However, a different *T. brucei* parasite strain (TREU 667 strain) causing a more chronic infection involving also revealed that the hepatosplenomegaly could contribute to hemodilution (140). Of note, also in HAT patients exhibiting anemia during later stages of the disease, hepatosplenomegaly has been recorded and this might therefore contribute to the observed “apparent” anemia (28). In this particular model, it seems that the virulence of the parasites determines whether hemodilution occurs. Interestingly, these observations correlate nicely with experimental *T. brucei* and *T. congolense* infections in domestic animals (cattle and sheep, respectively) (141, 142). Importantly, it was shown that MIF can also be present in erythrocytes and upon (hemo)lysis, due to oxidative stress, this factor can be released to further fuel inflammation (143). Given that hemolysis was shown to occur during *T. congolense* infections (40, 144), the increased levels of MIF observed during both the acute and chronic stage might mainly be due to parasite-inflicted rather that host-mediated damage of RBCs, which could also fuel a chronic (low-grade) anemia profile. Therefore, MIF might be an “important” player (upstream regulator) in African trypanosomiasis-associated anemia, mainly during the chronic stage of anemia development by fueling/promoting pathogenic M1 and could be considered as a prime anti-disease target.

## General Conclusion and Perspectives

African trypanosomes are very proficient in sculpturing a temporal environment to allow a gradual parasite establishment (27). Hereby, the host’s response at different stages of the infection determines whether pathogenicity/anemia develops. The mechanisms underlying African trypanosomiasis-associated anemia are multifactorial, whereby various molecules influence differentially the progression/development of anemia at distinct stages of infection. Initially, acute anemia seems to develop as part of the innate immune response upon infection, where parasite-derived factors, such as parasite-derived EVs, as well as host-derived (parasite-induced) IFN-γ trigger M1 cell differentiation that in turn produce pro-inflammatory molecules to control the infection. In this context, IFN-γ produced at the acute stage is the driving factor leading to acute/hemophagocytic anemia (30). In addition, the release of parasite-derived EVs in concert with host-derived TNF-α affect RBC survival and thereby fuels RBC elimination trough erythrophagocytosis (Figure 2). This is followed by a partial recovery, mediated most likely *via* extramedullary erythropoiesis, as a homeostatic reaction and a transient IL-10 production to dampen the pathogenic effects of the M1. Depending on the level of insult (i.e., M1-induced damage) and the capacity of the host to trigger and subsequently maintain IL-10 production, anemia is either alleviated (i.e., trypanotolerant animals) or sustained (i.e., trypanosusceptible animals). At this stage, MIF is an important host-derived factor determining/regulating the progression of anemia by promoting a persistent pro-inflammatory immune response and suppressing erythropoiesis. In addition, IFN-γ, TNF-α, and MIF are important molecules exerting a negative effect on erythropoiesis and at the same time at promoting erythrophagocytosis. By contrast, IL-10 was shown to positively affect erythropoiesis by downregulating the effects of the pro-inflammatory cytokines (145). Therefore, the balance between these pro- and anti-inflammatory cytokines during the course of infection determines the course of anemia development (51). In other words, the ability of the host to mount an efficient erythropoietic response (stress-induced response) to compensate for the enhanced erythrophagocytosis determines whether anemia persists. In the murine model, but also in cattle, the erythropoietic potential determines the level of anemia (33, 40, 94, 122). However, it seems that chronic anemia is most likely host-inflicted and due to a disproportional immune response (30). In summary, the mechanisms underlying/promoting chronic anemia development during *T. brucei* and *T. congolense* infections seem to be different. In the *T. brucei* infection model the main driving forces for anemia development are (i) the persistence of M1 that promote enhanced RBC elimination and iron retention and (ii) an insufficient erythropoiesis due to iron deprivation and the presence of pro-inflammatory cytokines that suppress RBC differentiation/maturation (Figure 3). By contrast, in the *T. congolense* infection model these aspects seem to play a main role only during the acute stage, as once M2 are induced and expanding they can in concert with IL-10 dampen to a certain extent the pathogenic effects of the M1. Given that *T. congolense*-infected animals still exhibited chronic anemia despite the presence of M2 and IL-10 suggests that the underlying mechanisms of chronic anemia in this model might be different and might rely on the hematopoietic potential of the animals (40). It was proposed that the month-lasting low-grade inflammatory response can also drive erythrophagocytosis, where the ensuing catabolism of hemoglobin resulted in iron accumulation mainly in the spleen and is followed by the enhanced release of bilirubin in the blood circulation (94). The resulting hyperbilirubinemia could favor the externalization of phosphatidylserine on RBCs and thus further contribute to erythrophagocytosis or eryptosis during *T*. *congolense* infection (146). However, at this stage the persistence of IL-10 might be a double-edged sword by on one hand dampening tissue injury and reduce the suppression on erythropoiesis mediated by the M1-released pro-inflammatory mediators and on the other promoting (i) ferritin expression thereby indirectly affecting iron availability, (ii) thrombocytopenia, and (iii) splenomegaly, leading to hemodilution (147, 148). The latter might in turn culminate into the occurrence of apparent anemia development, despite an enhanced erythropoiesis activity. In this context, it was shown that sustained secretion of IL-10 from transduced muscle leads to thrombocytopenia and splenomegaly in mice injected with rAAV1-IL-10 (147). Interestingly, thrombocytopenia was also documented for *T. congolense*-infected animals (149, 150). The splenomegaly observed in both susceptible and tolerant animals is required to accommodate the increased demand for erythropoiesis (93–95). However, it seems that this excessive accumulation of immature RBCs is most likely due to an inefficient erythropoietic potential (e.g., iron retention or unresponsiveness toward EPO or inefficient functioning of erythroblastic islands). Indeed, it was shown that most genes involved in erythropoiesis were found to be significantly modulated during the course of both *T. brucei* and *T. congolense* infection (40, 93, 94). However, so far information regarding erythropoiesis during African trypanosomiasis is limited and requires more attention. Also, a possible involvement of macrophages (functionality) within erythroblastic islands requires consideration. Within the era of genomics/proteomics (151–154), we can assume that novel pathways, mechanisms and molecular target molecules will be identified in various mouse models (155, 156). These discoveries will help us to refine our understanding of the mechanisms underlying anemia development and could even pave the way to develop new intervention strategies to alleviate it.

## Author Contributions

All authors listed have made a substantial, direct, and intellectual contribution to the work and approved it for publication.

## Conflict of Interest Statement

The authors declare that the research was conducted in the absence of any commercial or financial relationships that could be construed as a potential conflict of interest.
